# Presurgical Identification of Uterine Smooth Muscle Malignancies through the Characteristic FDG Uptake Pattern on PET Scans

**DOI:** 10.1155/2018/7890241

**Published:** 2018-06-19

**Authors:** Kung-Chu Ho, Yu-Hua Dean Fang, Gigin Lin, Shir-Hwa Ueng, Tzu-I Wu, Chyong-Huey Lai, Ho-Yen Chueh, Angel Chao, Ting-Chang Chang, Tzu-Chen Yen

**Affiliations:** ^1^Department of Nuclear Medicine, Chang Gung Memorial Hospital and Chang Gung University, Keelung, Taiwan; ^2^Department of Biomedical Engineering, National Cheng Kung University, Tainan, Taiwan; ^3^Department of Medical Imaging and Intervention, Chang Gung Memorial Hospital and Chang Gung University, Taoyuan, Taiwan; ^4^Department of Pathology, Chang Gung Memorial Hospital and Chang Gung University, Taoyuan, Taiwan; ^5^Department of Obstetrics and Gynecology, Wan Fang Hospital, Taipei Medical University, Taipei, Taiwan; ^6^Department of Obstetrics and Gynecology, Chang Gung Memorial Hospital and Chang Gung University, Taoyuan, Taiwan; ^7^Department of Nuclear Medicine and Center for Advanced Molecular Imaging and Translation, Chang Gung Memorial Hospital and Chang Gung University, Taoyuan, Taiwan; ^8^Department of Nuclear Medicine, Xiamen Chang Gung Hospital, Fujian, China

## Abstract

The unidentified presence of uterine smooth muscle malignancies poses a tremendous risk in women planning surgery for presumed benign leiomyomas. We sought to investigate whether preoperative FDG PET may be useful to identify leiomyosarcomas (LMS) and smooth muscle tumors of uncertain malignant potential (STUMP). *Methods*. We investigated patients with rapidly growing uterine masses which were suspected of being malignant on ultrasound or MRI. Among the 21 patients who underwent FDG PET, we identified 7 LMS, 1 STUMP, and 13 leiomyomas. PET-derived parameters and FDG uptake patterns were analyzed retrospectively. *Results*. The SUV_max_ values of LMS/STUMP (range: 3.7–11.8) were significantly higher than those observed in leiomyomas (range: 2.0–9.4; *P*=0.003) despite a significant overlap. The metabolic tumor/necrosis ratio was significantly higher in LMS/STUMP than in leiomyomas (*P* < 0.001), with no significant intergroup overlaps. All LMS/STUMP revealed a characteristic pattern of FDG uptake, identifying a specific “hollow ball” sign (corresponding to areas of coagulative tumor necrosis). In contrast, this sign was invariably absent in patients with leiomyomas. *Conclusion*. The characteristic FDG uptake pattern instead of SUV on PET images allows identifying LMS/STUMP in patients with rapidly growing uterine masses, avoiding the deleterious consequences of regular surgery for presumed benign leiomyomas.

## 1. Introduction

The distinction between uterine leiomyosarcomas (LMS) and benign uterine leiomyomas remains challenging because the two conditions share similar clinical symptoms (i.e., abnormal uterine bleeding, pelvic pain, and/or abdominal bloating) [1]. Because uterine LMS originates from the myometrium, endometrial sampling in LMS has limited clinical utility. In a recent study, only 24 of the 68 (35%) patients who were diagnosed with uterine LMS on final pathology were correctly identified preoperatively through endometrial sampling (either using pipelle biopsy or dilation and curettage) [2]. The correct identification of a uterine LMS may be problematic in patients with rapidly growing uterine mass [1, 3]. Notably, uterine morcellation during minimally invasive surgery has been associated with worse survival outcomes in patients with undiagnosed LMS and is discouraged by the U.S. Food and Drug Administration [4].

The diagnosis of uterine LMS is generally obtained following a myomectomy or a hysterectomy performed for a purportedly benign disease [5]. The histopathological criteria for diagnosing uterine LMS include the presence of moderate-to-severe cytologic atypia, a high mitotic index (≥10 per 10 high-power field), and evidence of coagulative tumor cell necrosis. Uterine smooth muscle tumors of uncertain malignant potential (STUMP) share certain characteristics with LMS, albeit not meeting all of the abovementioned diagnostic criteria [6, 7].

Despite multiple studies in the field [5, 8], no reliable preoperative imaging modality to differentiate benign from malignant uterine tumors has been identified yet. On ultrasound, LMS may present as heterogeneous masses with bizarre internal echo patterns. Unfortunately, imaging findings of LMS and benign leiomyomas may be largely overlapping [9]. On MRI, uterine LMS/STUMP can display high T1-weighted (because of hemorrhage) and T2-weighted (because of necrosis) signals [10, 11]. Unfortunately, a clear-cut distinction between LMS/STUMP and benign leiomyomas on MRI remains problematic because of atypical imaging features [12].

FDG PET is clinically useful for the staging of uterine sarcoma [13]. Although there are differences in terms of standardized uptake value (SUV) between uterine LMS and leiomyomas, its diagnostic accuracy for LMS is as low as 73% [14]. In addition, the discrimination between LMS and leiomyomas may be hampered by the small differences in FDG uptake (which is generally high in the former and mild in the latter) between the two conditions [15]. In patients with positive or equivocal findings on FDG PET, the use of 16*α*-[18F]-fluoro-17*β*-oestradiol PET may provide additional information for distinguishing between LMS and leiomyomas [16].

It has been recently shown that contrast-enhanced MRI can accurately distinguish between uterine LMS/STUMP and benign leiomyomas by the findings of central nonenhancement specifically reflecting the presence of necrotic areas within the tumor [17]. This observation led us to the hypothesis that the metabolic characteristics of LMS/STUMP (reflected by the presence of coagulative tumor cell necrosis) could be more useful than SUV for diagnostic purposes.

Starting from these premises, we designed the current study to investigate whether the patterns of FDG uptake corresponding to areas of coagulative tumor cell necrosis on PET images can be helpful in distinguishing between LMS/STUMP and benign leiomyomas during the preoperative period.

## 2. Materials and Methods

### 2.1. Patients

Eligibility criteria for undergoing FDG PET were as follows: (1) doubling of the perpendicular lesion diameter over a 3/6-month period, with a tumor diameter >10 cm on ultrasound or MRI (in cases without clinical symptoms) or >5 cm (in cases with clinical symptoms), regardless of the menopausal status; (2) presence of a tumor with a diameter >5 cm on ultrasound or MRI in a postmenopausal woman; and (3) any of the following two conditions: (a) suspected malignancy showing a heterogeneous and bizarre echo pattern on ultrasound and (b) suspected malignancy showing a high signal intensity on T1- and T2-weighted images on MRI. Patients with biopsy-proven endometrial malignancies different from LMS/STUMP (i.e., endometrial cancer, carcinosarcoma, endometrial stromal sarcoma, or adenosarcoma) were excluded. We also excluded patients who were unable to provide informed consent and/or had any contraindication for surgery. This is a single-institution study, and the Institutional Review Board of the Chang Gung Memorial Hospital approved the study protocol (IRB 97-2366B). Written informed consent was obtained from all participants.

### 2.2. MRI Protocol and Image Analysis

MR images were acquired using a 3.0 T scanner (Trio Tim, Siemens Medical Systems, Erlangen, Germany). The acquisition protocol has been previously described in detail [17]. T1- and T2-weighted images were used for selecting suitable candidates for this study.

### 2.3. FDG PET Image Acquisition

Patients were asked to fast for at least 4 h before examination and were required to have a blood glucose level <200 mg/dL. No intravenous contrast enhancement was used. Patients were injected intravenously with 370–555 MBq ^18^F-FDG (depending on body weight), and images were acquired 60 min after its administration. Whole-body PET emission scans were obtained from the base of the skull to the midthigh, without position changes. FDG PET/CT was performed on a Discovery ST 16 scanner (GE Healthcare, Milwaukee, WI, USA). Low-dose CT images were used for attenuation correction of PET data. PET images were reconstructed using a CT-based attenuation correction with an ordered-subset expectation maximization iterative reconstruction algorithm (4 iterations and 10 subsets). When these reconstruction parameters were used, the axial spatial resolution of PET at the center of the gantry was 4.80 mm. The scanner underwent 3D normalization well counter correction every three months for optimizing its quantitative accuracy.

### 2.4. FDG PET Imaging Analysis

PET images were analyzed on a dedicated workstation (Xeleris 3.0; GE Healthcare). The SUV for each voxel was calculated as follows: SUV = (measured activity concentration (Bq/mL))/(injected activity (Bq)/body weight (kg) × 1,000). The maximum SUV (SUV_max_) was measured as the maximum value of SUV in each voxel within the volume of interest drawn on the uterine tumor.

**Figure 1:**
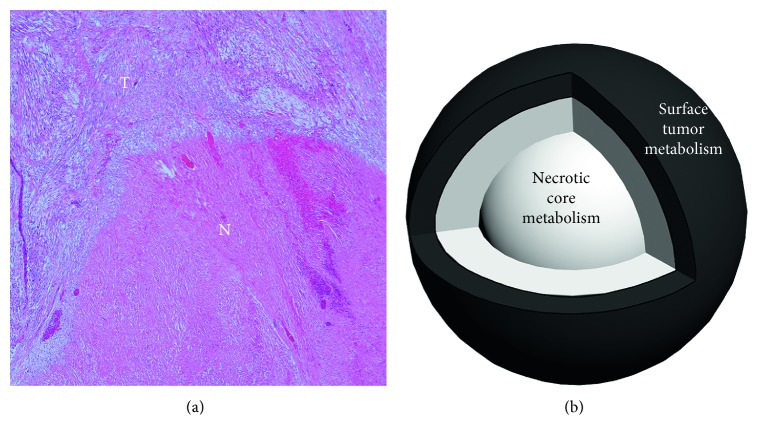
(a) The histopathological examination of a leiomyosarcoma revealed an abrupt transition between the area of coagulative tumor cell necrosis (letter N) and viable, well-preserved tumor cells (letter T) (hematoxylin and eosin staining, 40x magnification). (b) Schematic representation of the newly developed PET parameter (metabolic tumor/necrosis ratio) used in this study for uterine tumors. The metabolic tumor/necrosis ratio was defined as the ratio between surface tumor metabolism and the necrotic core metabolism.

A specific PET imaging parameter termed “metabolic tumor/necrosis ratio” was developed to quantify the presence of coagulative tumor cell necrosis in LMS/STUMP (Figure 1(a)) and calculated with an automated approach (Figure 1(b)) as follows:

(1) $$\begin{array}{c} \frac{\mathrm{metabolic}\;\mathrm{tumor}}{\mathrm{necrosis}\;\mathrm{ratio}}=\frac{\mathrm{surface}\;\mathrm{tumor}\;\mathrm{metabolism}}{\mathrm{necrotic}\;\mathrm{core}\;\mathrm{metabolism}}. \end{array}$$

The procedure for determining “necrotic core metabolism” was as follows. We initially reasoned that tumors were heterogeneous and the necrotic core was not invariably located in the central area but could also be positioned eccentrically within the tumor. We therefore used the intensity-weighted gradient magnitude image to segment the necrotic core (characterized by a low gradient magnitude and a low intensity). First, the volume of interest (VOI) for the lesion was drawn semiautomatically using a SUV threshold of 2.5 (denoted with the lesion mask M1). The lesion volume was then segmented out of the image data. Second, we sought to enhance the intensity contrast. To this aim, the voxel intensities within the lesion were redigitized into 16 equally spaced bins with the minimum and maximum postdigitalization intensities mapped to zero and fifteen, respectively. Third, the three-dimensional gradient magnitude was calculated with the redigitized intensities and subsequently multiplied by the original image intensities to provide the intensity-weighted gradient magnitude image (which was in turn smoothed using a 3-by-3-by-3 averaging kernel and finally eroded once). The voxel with the minimal value within the intensity-weighted gradient magnitude image was identified and used as a seed point for region growing. To achieve this goal, its tripled intensity was considered as the region-growing threshold. The segmented results obtained up to this point were regarded as the “necrotic core VOI.” The mean redigitized intensity from the “necrotic core VOI” was termed “necrotic core metabolism.”

The “surface tumor VOI” was the prerequisite for calculation of the “surface tumor metabolism.” Its segmentation was performed as follows. First, we calculated the parameter *k* with the formula:

(2) $$\begin{array}{c} k=\sqrt[{3}]{n}\;*\;0.1, \end{array}$$

where *n* indicates the total number of voxels within the entire lesion VOI. The value of *k* was rounded to the nearest integer because it was proportional to the VOI thickness. Second, upon determination of *k*, a novel M2 mask was obtained through the erosion of the original M1 mask for *k* times using the previously employed kernel. The “surface tumor VOI” was determined by subtracting M2 from M1. Finally, the mean redigitized intensity from the “surface tumor VOI” was defined as the “surface tumor metabolism.”

### 2.5. Statistical Analysis

SUV_max_ values and metabolic tumor/necrosis ratios of patients with LMS/STUMP versus those with benign leiomyomas were compared with the Mann–Whitney *U* test. The association between SUV_max_ and mitotic count in LMS/STUMP was analyzed with Pearson's correlation coefficient. The optimal cutoff for SUV_max_ was identified by receiver operating characteristic (ROC) analysis. McNemar's test was used to compare the sensitivity, specificity, and diagnostic accuracy between ultrasound, contrast-enhanced MRI, and FDG PET. All calculations were performed with the SPSS 18.0 statistical package (SPSS Inc., Chicago, IL, USA). *P* values < 0.05 (two-tailed) were considered statistically significant.

## 3. Results

### 3.1. Patients

Between 2004 and 2013, we identified a total of 21 patients who were eligible for FDG PET (Table 1). All patients were treated with surgery upon completion of all imaging studies. The final histopathological diagnosis was LMS in seven patients, STUMP in one case, and benign leiomyomas in 13 patients. The median age was 48 years, and the median tumor size was 10 cm in both the LMS/STUMP and leiomyoma groups. The general characteristics of the study patients are summarized in Table 1.

### 3.2. PET Imaging Parameters in the LMS/STUMP versus Leiomyoma Groups

**Figure 2:**
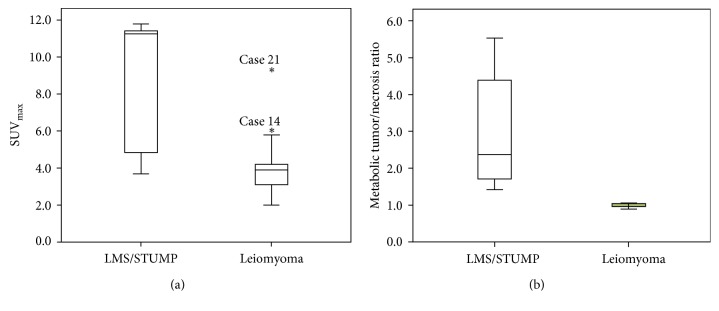
Box-and-whisker plots of SUV_max_ in the leiomyosarcomas (LMS) and smooth muscle tumors of uncertain malignant potential (STUMP) group compared with patients with benign leiomyomas. (a) The SUV_max_ in the LMS/STUMP group was significantly higher than that observed in the leiomyoma group (*P*=0.003). However, SUV_max_ values showed a significant intergroup overlap (the case numbers reported in (a) correspond to those in Table 2). (b) The metabolic tumor/necrosis ratio in the LMS/STUMP group was significantly higher than that observed in patients with leiomyomas (*P* < 0.001); notably, no overlap was observed between the two groups.

SUV_max_ was significantly higher in the LMS/STUMP group compared with patients with leiomyomas (*P*=0.003). However, the range of SUV_max_ values in the LMS/STUMP group (3.7–11.8) showed a large overlap with those observed in patients with leiomyomas (2.0–9.4; Figure 2(a)). We then profiled the metabolic characteristics of uterine tumors using a newly developed PET parameter termed “metabolic tumor/necrosis ratio.” Unfortunately, we were unable to reanalyze the images of seven patients (whose raw data were lost because of a >10-year storage time). The metabolic tumor/necrosis ratio was significantly higher in the LMS/STUMP group than in patients with leiomyomas (*P* < 0.001). Interestingly, no significant overlaps in terms of metabolic tumor/necrosis ratio were observed between the LMS/STUMP and leiomyoma groups (Figure 2(b)). We then attempted to clarify the relationship between metabolic activity and tumor aggressiveness in patients with LMS/STUMP. Remarkably, the SUV_max_ values were found to correlate significantly with the mitotic count (*r*=0.840, *P*=0.009; Table 2).

### 3.3. FDG Uptake Patterns in the LMS/STUMP versus Leiomyoma Groups

**Figure 3:**
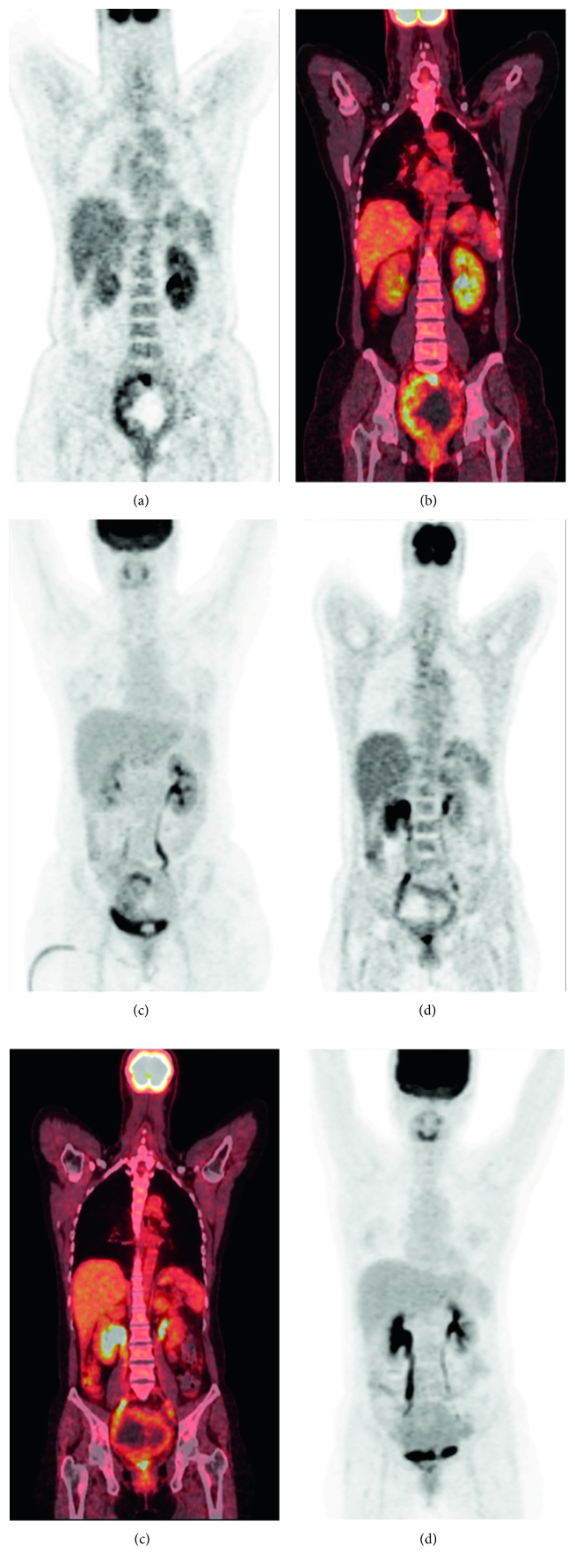
Illustrative images of two patients with leiomyosarcomas (LMS) (the case numbers reported in Figure 3 correspond to those in Table 2). Case #1 had a high-grade LMS and showed the “hollow ball” sign on the coronal view of the FDG PET image (a), PET/CT fusion image (b), and maximum-intensity projection image (c). Case #6 had a low-grade LMS and showed the “hollow ball” sign on the coronal view of the FDG PET image (d), PET/CT fusion image (e), and maximum-intensity projection image (f).

**Figure 4:**
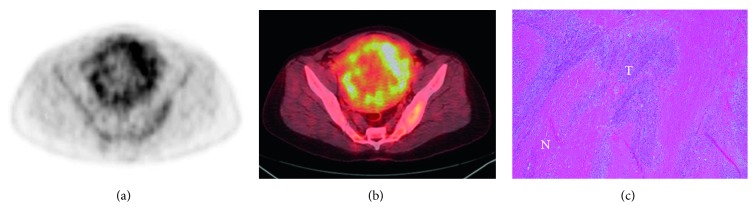
Illustrative images of a patient with a smooth muscle tumor of uncertain malignant potential (STUMP). The “hollow ball” sign was evident on the transaxial view of FDG PET image (a) and PET/CT fusion image (b). The histopathological examination revealed foci of necrosis (letter N) among tumor cells (letter T) (hematoxylin and eosin staining, 100x magnification) (c).

**Figure 5:**
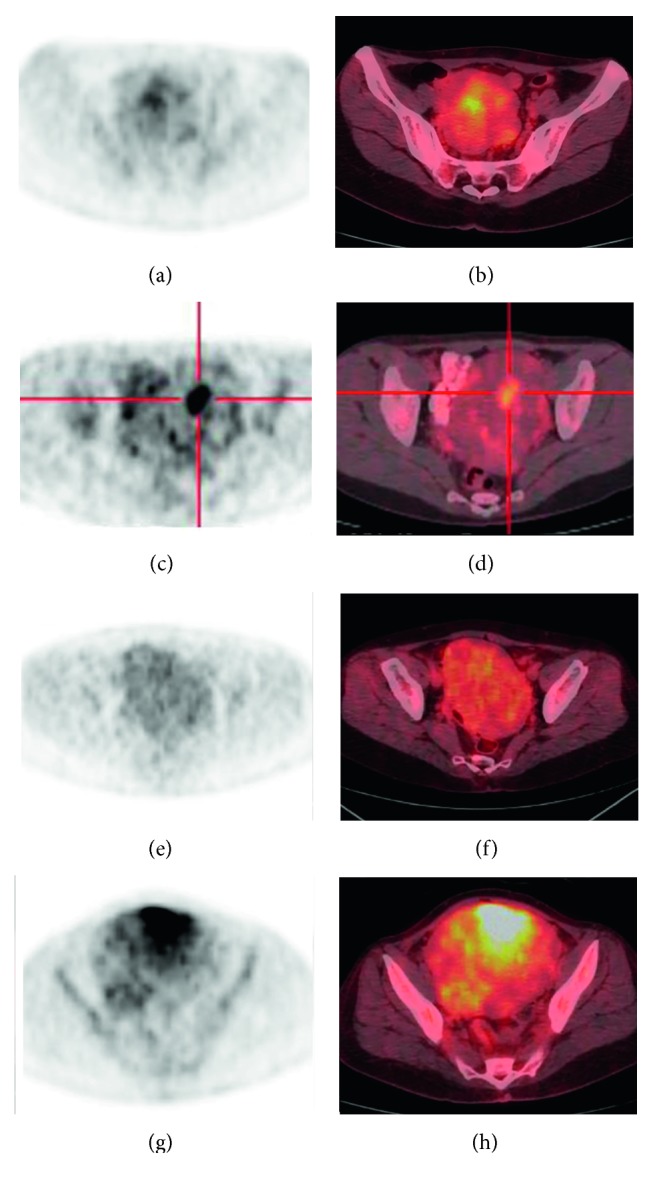
Illustrative images of four FDG uptake patterns in benign leiomyomas (the case numbers reported in Figure 5 correspond to those in Table 2). A heterogeneous FDG uptake pattern in case #13 was evident on the transaxial view of the FDG PET image (a) and PET/CT fusion image (b). A focal FDG uptake pattern in case #16 was identified on the transaxial view of the FDG PET image (c) and PET/CT fusion image (d). A diffuse low FDG uptake pattern in case #18 was evident on the transaxial view of the FDG PET image (e) and PET/CT fusion image (f). A diffuse high FDG uptake pattern in case #21 was identified on the transaxial view of the FDG PET image (g) and PET/CT fusion image (h).

The FDG uptake pattern was superior to SUV_max_ in distinguishing the LMS/STUMP group from patients with leiomyomas on preoperative PET images. In line with the clinical utility of the metabolic tumor/necrosis ratio in identifying the presence of tumor necrosis, the LMS/STUMP group was characterized by the presence of the “hollow ball” sign on their FDG PET images (Figures 3 and 4). Notably, all of the patients with high-grade LMS (*n*=5), low-grade LMS (*n*=2), and STUMP (*n*=1) showed the “hollow ball” sign, which was consistently absent in all cases with benign leiomyomas (Table 2). Eventually, the patterns of FDG uptake observed in the gross tumor mass of patients with leiomyomas were as follows: heterogeneous uptake (*n*=4), focal uptake (*n*=4), diffuse high uptake (*n*=1), and diffuse low uptake (*n*=4; Figure 5).

### 3.4. Diagnostic Accuracy of PET Imaging Parameters and FDG Uptake Pattern

The presence of a “hollow ball” sign on FDG PET did not yield false-negative or false-positive results in any of the study patients. Identical results were obtained when the metabolic tumor/necrosis ratio was analyzed. In contrast, the use of the optimal cutoff point for SUV_max_ (4.5 based on the results of ROC analysis) produced false-positive findings in three cases and false-negative results in two patients (Table 3). The diagnostic accuracy of SUV_max_ was marginally lower than that of the FDG uptake pattern on PET images (*P*=0.063).

## 4. Discussion

LMS are generally >5 cm in size and are commonly characterized by the presence of necrotic and hemorrhagic areas [18]. For this reason, patients with rapidly growing uterine masses larger than 5 cm and suspected of being malignant on ultrasound or MRI were deemed eligible for FDG PET imaging in this study. Patients with other malignancies (e.g., endometrial cancer) were carefully excluded through endometrial biopsies or dilation and curettage. Standard MRI criteria for identifying hemorrhage and necrosis on T1- and T2-weighted images have been previously utilized for patient selection [10, 11]. The combined assessment of FDG PET and MRI images is superior to MRI alone in detecting uterine smooth muscle tumors [19]. In our study, SUV_max_ was significantly higher in the LMS/STUMP group compared with patients with leiomyomas; unfortunately, its clinical usefulness in distinguishing between LMS/STUMP and leiomyomas was hampered by the significant overlap between malignant and benign lesions. Notably, SUV_max_ values were found to be significantly correlated with the mitotic index. This observation suggests an association between glucose metabolism and tumor aggressiveness, which is in line with previous data showing worse clinical outcomes in patients with LSM characterized by high tumor SUV_max_ [20].

The spatial pattern of FDG uptake may offer diagnostic information beyond that provided by SUV_max_. For example, it has been previously shown that focal and diffuse FDG uptake patterns of pancreatic lesions were superior in terms of sensitivity and specificity to MRI and other PET-derived parameters (e.g., SUV_early_, SUV_delayed_, lesion-to-background ratio, and retention index) for differentiating benign from malignant pancreatic masses [21]. Because the presence of coagulative tumor cell necrosis is an essential diagnostic criterion for LMS/STUMP [18], we reasoned that a PET-derived metabolic parameter reflecting coagulative tumor necrosis would be extremely helpful for the presurgical identification of LMS/STUMP.

Histopathologically, coagulative tumor cell necrosis is characterized by an abrupt transition between necrotic and viable, well-preserved tumor cells. In contrast, hyaline necrosis occurring in leiomyoma shows a variable amount of hyalinized collagen interposed between the central degenerated region and peripheral preserved smooth muscle cells [18]. Here, we hypothesized that the abrupt edge between viable and necrotic tissue (which is typical of LMS/STUMP) would be paramount for their identification on FDG PET images. Actually, the typical FDG uptake pattern that reflected the presence of such lesions was the “hollow ball” sign. Remarkably, such sign was invariably absent in all of the patients with leiomyomas (because the hyaline necrosis which is typical of these benign lesions has no abrupt transitions inside).

Texture features of PET images—reflecting spatial heterogeneity in tumors—have been shown to provide useful prognostic information [22] but show poor correlations with histopathological features [23]. Herein, we developed a novel imaging parameter—termed metabolic tumor/necrosis ratio—which was specifically aimed at quantifying the metabolic characteristics of tumor necrosis in LMS/STUMP. However, segmentation of the surface tumor and the necrotic areas was challenging owing to tumor heterogeneity and the eccentric core. A complex multistep image processing was required for determining the metabolic tumor/necrosis ratio. Although our analysis was limited by the absence of raw data in seven patients, we believe that the metabolic tumor/necrosis ratio may serve as a quantitative confirmatory parameter for the presence of LMS/STUMP when the “hollow ball” sign is identified by visual assessment. This approach may be especially useful in the presence of an equivocal “hollow ball” sign.

All of the LMS/STUMP examined in this study showed areas of tumor necrosis. However, uterine smooth muscle tumors with moderate-to-severe atypia accompanied by a high mitotic index can also be histologically diagnosed as LMS/STUMP even in the absence of coagulative tumor cell necrosis [18]. We are aware that the absence of tumor necrosis would limit the diagnostic utility of the FDG uptake pattern for identifying certain LMS/STUMP. In general, we suggest that the presence of a “hollow ball” sign can confidently identify a lesion as LMS/STUMP. Conversely, we cannot exclude that the “hollow ball” sign can be absent in some LMS/STUMP because of the lack of tumor necrosis; this event did not occur in this study possibly because of the small sample size. Based on our small cohort, the “hollow ball” sign on FDG PET is a potentially unique sign to distinguish LMS/STUMP from benign leiomyomas. We expected that further prospective larger cohort studies conducted by Asian Gynecologic Oncology Group would help in confirming the diagnosis. As the availability of multimodality imaging systems such as PET/MRI improves, we are optimistic that the diagnostic discrimination between LMS/STUMP and benign leiomyomas will be ameliorated in the near future, ultimately exerting a positive impact on clinical management.

## 5. Conclusions

Our study shows that the presence of the “hollow ball” sign on FDG PET imaging allows distinguishing LMS/STUMP from benign leiomyomas in patients with rapidly growing large uterine masses. The metabolic tumor/necrosis ratio may be used as an additional confirmatory tool. Our findings have significant clinical implications and may ultimately avoid the deleterious consequences of regular surgery in patients with benign leiomyomas.

## Figures and Tables

**Table 1:** General characteristics of the study patients (*n*=21).

	LMS/STUMP		Benign leiomyoma	
Number of patients, *n* (%)	8 (38%)		13 (62%)	
Age (years)				
Median (range)	48 (42–81)		48 (32–54)	
Tumor size (cm)				
Median (range)	10.1 (5.5–17.4)		10.2 (4.5–16.7)	
Pathology, *n*	LMS	7	Ordinary leiomyoma	9
	STUMP	1	Degenerated leiomyoma	2
			Cellular leiomyoma	1
			Infarcted leiomyoma	1
Pathological staging, *n*	T1bN0M0	5		
	T2aN0M0	1		
	T2bN0M0	1		
	T1bN1M1	1		
Primary surgery, *n* (%)	ATH + BSO	8	ATH	12
			Hysteroscopic hysterectomy	1

LMS, leiomyosarcoma; STUMP, smooth muscle tumor with uncertain malignant potential; ATH, abdominal total hysterectomy; BSO, bilateral salpingo-oophorectomy.

**Table 2:** Detailed histopathological and imaging findings of the study patients (*n*=21).

Patient #	Pathological diagnosis	Metabolic tumor/necrosis ratio^*∗*^	SUV_max_	FDG uptake pattern	Tumor diameter^*∗∗*^ (cm)	Mitotic count (per 10 HPF)
1	High-grade LMS	4.388	11.1	“Hollow ball” sign	11.9	23
2	High-grade LMS	2.703	11.4	“Hollow ball” sign	5.5	25
3	High-grade LMS	1.707	11.4	“Hollow ball” sign	17.4	30
4	High-grade LMS	N/A	11.4	“Hollow ball” sign	11.4	20
5	High-grade LMS	N/A	11.8	“Hollow ball” sign	5.8	15
6	Low-grade LMS	5.533	4.3	“Hollow ball” sign	8.8	5
7	Low-grade LMS	1.421	3.7	“Hollow ball” sign	8.6	10
8	STUMP	2.036	5.4	“Hollow ball” sign	13.7	5
9	Ordinary leiomyoma	1.063	2.2	Diffuse low	14.0	
10	Ordinary leiomyoma	1.013	4.2	Focal	6.5	
11	Ordinary leiomyoma	0.964	4.0	Heterogeneous	8.8	
12	Ordinary leiomyoma	0.955	2.0	Diffuse low	16.0	
13	Ordinary leiomyoma	0.896	3.1	Heterogeneous	5.7	
14	Ordinary leiomyoma	N/A	6.0	Focal	10.2	
15	Ordinary leiomyoma	N/A	5.8	Heterogeneous	5.4	
16	Ordinary leiomyoma	N/A	3.9	Focal	5.0	
17	Ordinary leiomyoma	N/A	3.5	Focal	16.7	
18	Degenerated leiomyoma	1.038	2.5	Diffuse low	10.2	
19	Degenerated leiomyoma	N/A	3.5	Heterogeneous	11.6	
20	Cellular leiomyoma	1.069	2.5	Diffuse low	7.9	
21	Infarcted leiomyoma	1.034	9.4	Diffuse high	11.1	

^*∗*^The images of seven patients (whose raw data were lost because of a >10-year storage time) were not available for reanalysis; ^*∗∗*^measured on ultrasound or MRI; LMS, leiomyosarcoma; STUMP, smooth muscle tumor with uncertain malignant potential; N/A, not available; HPF, high-power field.

**Table 3:** Comparison of PET imaging parameters and FDG uptake pattern in distinguishing between LMS/STUMP and benign leiomyomas.

	TP	TN	FP	FN	N/A	Accuracy (%)	Sensitivity (%)	Specificity (%)	PPV (%)	NPV (%)
SUV_max_	6	10	3	2	0	76^*∗*^	75	77	67	83
Metabolic tumor/necrosis ratio	6	8	0	0	7	100	100	100	100	100
“Hollow ball” sign	8	13	0	0	0	100	100	100	100	100

^*∗*^ *P*=0.063, McNemar's test versus “hollow-ball” sign; LMS, leiomyosarcoma; STUMP, smooth muscle tumor with uncertain malignant potential; TP, true-positive; TN, true-negative; FP, false-positive; FN, false-negative; N/A, not available; PPV, positive predictive value; NPV, negative predictive value.

## Data Availability

The raw imaging data used to support the findings of this study are available from the corresponding author upon request.
